# Echocardiographic Evaluation of Hemodynamics in Neonates and Children

**DOI:** 10.3389/fped.2017.00201

**Published:** 2017-09-15

**Authors:** Yogen Singh

**Affiliations:** ^1^Department of Neonatology and Pediatric Cardiology, Cambridge University Hospitals NHS Foundation Trust, Cambridge, United Kingdom; ^2^University of Cambridge Clinical School of Medicine, Cambridge, United Kingdom

**Keywords:** hemodynamic evaluation, functional echocardiography, hemodynamic assessment in intensive care, echocardiography in NICU, neonates and children

## Abstract

Hemodynamic instability and inadequate cardiac performance are common in critically ill children. The clinical assessment of hemodynamic status is reliant upon physical examination supported by the clinical signs such as heart rate, blood pressure, capillary refill time, and measurement of the urine output and serum lactate. Unfortunately, all of these parameters are surrogate markers of cardiovascular well-being and they provide limited direct information regarding the adequacy of blood flow and tissue perfusion. A bedside point-of-care echocardiography can provide real-time hemodynamic information by assessing cardiac function, loading conditions (preload and afterload) and cardiac output. The echocardiography has the ability to provide longitudinal functional assessment in real time, which makes it an ideal tool for monitoring hemodynamic assessment in neonates and children. It is indispensable in the management of patients with shock, pulmonary hypertension, and patent ductus arteriosus. The echocardiography is the gold standard diagnostic tool to assess hemodynamic stability in patients with pericardial effusion, cardiac tamponade, and cardiac abnormalities such as congenital heart defects or valvar disorders. The information from echocardiography can be used to provide targeted treatment in intensive care settings such as need of fluid resuscitation versus inotropic support, choosing appropriate inotrope or vasopressor, and in providing specific interventions such as selective pulmonary vasodilators in pulmonary hypertension. The physiological information gathered from echocardiography may help in making timely, accurate, and appropriate diagnosis and providing specific treatment in sick patients. There is no surprise that use of bedside point-of-care echocardiography is rapidly gaining interest among neonatologists and intensivists, and it is now being used in clinical decision making for patients with hemodynamic instability. Like any other investigation, it has certain limitations and the most important limitation is its intermittent nature. Sometimes acquiring high quality images for precise functional assessment in a ventilated child can be challenging. Therefore, it should be used in conjunction with the existing tools (physical examination and clinical parameters) for hemodynamic assessment while making clinical decisions.

## Introduction

The goal of hemodynamic monitoring is to assess the adequacy of perfusion and tissue oxygenation, which primarily depends upon preload, cardiac function, afterload, and adequate perfusion pressure. The physiological information from hemodynamic monitoring allows the clinicians in making early diagnosis and providing specific treatment. The clinical assessment of hemodynamic status is reliant upon monitoring heart rate (HR), capillary refill time, serum lactate, urine output, and often continuous monitoring of blood pressure (BP). These parameters are only surrogate markers of the adequacy of tissue oxygenation and perfusion, and are poorly validated for cardiovascular assessment in children with hemodynamic instability (1). Moreover, there is a lack of consensus on “normal BP” in neonates. This becomes even more challenging in preterm infants and sick neonates, when complex hemodynamic changes occur with rapid changes in the pulmonary and systemic vascular resistances in first few weeks after birth. Without a good understanding of these physiological changes, providing a high quality care to critically ill neonates with cardiovascular compromise may be challenging, and it can lead to incorrect assumptions or incorrect therapeutic interventions (1–6).

The echocardiography is increasingly being used for hemodynamic assessment and monitoring in the intensive care units (ICUs), and with the use of bedside echocardiography, it is possible to assess cardiac function, preload, afterload, fluid responsiveness and estimate pulmonary artery pressure (PAP) and cardiac output. Echocardiography is easily available on beside, it is non-invasive and portable, and it can be used to acquire information of physiological changes in real time. This physiological information, in conjunction with clinical assessment, can help in guiding targeted specific therapy (7–9).

The role of functional echocardiography is evolving at a rapid pace, and currently, it is considered a key tool for hemodynamic assessment in the intensive care setting. Recently, published studies have demonstrated significance of the use of echocardiography in management of critically ill patients. The studies have reported that clinical management was changed in 30–60% cases after point-of-care echocardiography, and now it is considered as an indispensable tool in management of shock (10, 11). The expert consensus statement on management of shock in children has emphasized the importance of echocardiography in identifying underlying pathophysiology, and categorization of shock as distributive, hypovolemic, obstructive, or cardiogenic (12). In the neonatal and pediatric population, the use of echocardiography becomes even more important because clinical parameters used for hemodynamic assessment are ineffective and unreliable.

Hence, it is no surprise that there has been a significant increase in the use of bedside echocardiography in the neonatal and pediatric ICUs. Different terms have been used to describe the use of bedside echocardiography in various settings such as neonatologist-performed echocardiography, targeted neonatal echocardiography, functional echocardiography, or point-of-care-ultrasound (7–9). However, the purpose remains the same—to acquire the physiological information of hemodynamic well-being and use this information in clinical decision making for sick children. Despite its increasing popularity and importance in ICU setting, there is no established structured training program for neonatologists or intensivists to acquire echocardiography skills (7–9, 13–18). At least three expert consensus statements have been published in the last 5 years highlighting the urgent need of a structured training program and accreditation process specifically tailored for the neonatologists and intensivists (7–9).

In this article, we will review the role of echocardiography in hemodynamic evaluation in neonates and children, and re-visit various echocardiographic parameters which could be helpful specifically in ICU setting. At this point, it is worth emphasizing on using ECG during performing echocardiography, especially while functional assessment.

## Assessment of Preload and Fluid Responsiveness

The accurate estimation of preload on echocardiography is one of the most challenging areas. This becomes even more difficult in neonates and young children needing mechanical ventilation, which has a varying effect on loading conditions (filling) and cardiac function. Moreover, changing lung compliance complicates the pressure–volume relationship even further (19). The assessment of preload on bedside echocardiography is performed by examining the left ventricle (LV), inferior vena cava (IVC), and the right heart. Preloading assessment can be reliably made on “eyeballing” the heart in the apical four-chamber view in expert hands (qualitative assessment). The quantitative assessment involves measuring left ventricular volumes and collapsibility index of the IVC.

The left ventricular end-diastolic area and volume are probably best measured by using the Simpson’s biplane method (20). The latter requires identification of endocardial border, which can be challenging in neonates and young children requiring mechanical ventilation. In emergency situations, a simpler approach may be used: obliteration of the LV cavity, also known as “kissing ventricles” suggests hypovolemia.

Generally, right ventricular dimensions are normally smaller than those of the LV in children. The dilatation of right ventricle (RV) may indicate volume overloading of right side of the heart or pulmonary hypertension, and to complicate the matter even further, neonates may have slightly dominant RV soon after birth (physiological RV dominance). Similarly, an enlarged right atrium (RA) with bowing of intra-atrial septum toward the left atrium may indicate elevated right atrial pressure.

The triad of a “kissing” small LV cavity, RV size, and a normal or small RA is strongly suggestive of hypovolemia. However, this is relevant only in spontaneously breathing patients and not applicable to children needing mechanical ventilation or abdominal distension.

One of the major challenges in the intensive care setting is to recognize how a particular child is going to respond to fluid therapy—who will improve, who would have no response, and who can even deteriorate with a fluid bolus? Apart from “eyeballing” for under-filled heart (qualitative assessment), the following quantitative methods have been described to assess the fluid responsiveness in children.

### Variation in Left Ventricular Outflow Tract Velocity Time Integral (VTI)

The variation in VTI at left ventricular outflow tract level or just proximal to aortic valve (AV) level during inspiration and expiration has been reported to predict volume responsiveness. A variation of >15% has been reported to have a high predictive value with a sensitivity and specificity exceeding 90% (21, 22). This has been validated in several studies, including many studies involving children needing mechanical ventilation (23–31), and it seems a promising echocardiographic parameter to assess fluid responsiveness in children.

### IVC Collapsibility Index

The IVC collapsibility index is calculated by measuring the maximum (Dmax) and minimum IVC diameter (Dmin) from the subcostal view on echocardiography, and a collapsibility of >55% is reported to be predictive of fluid responsiveness (32, 33):

$$\text{IVCDI}=\frac{\text{Dmax}-\text{Dmin}}{\text{Dmax}}\times 100\%.$$

### IVC Distensibility Index (IVCDI)

**Figure 1:**
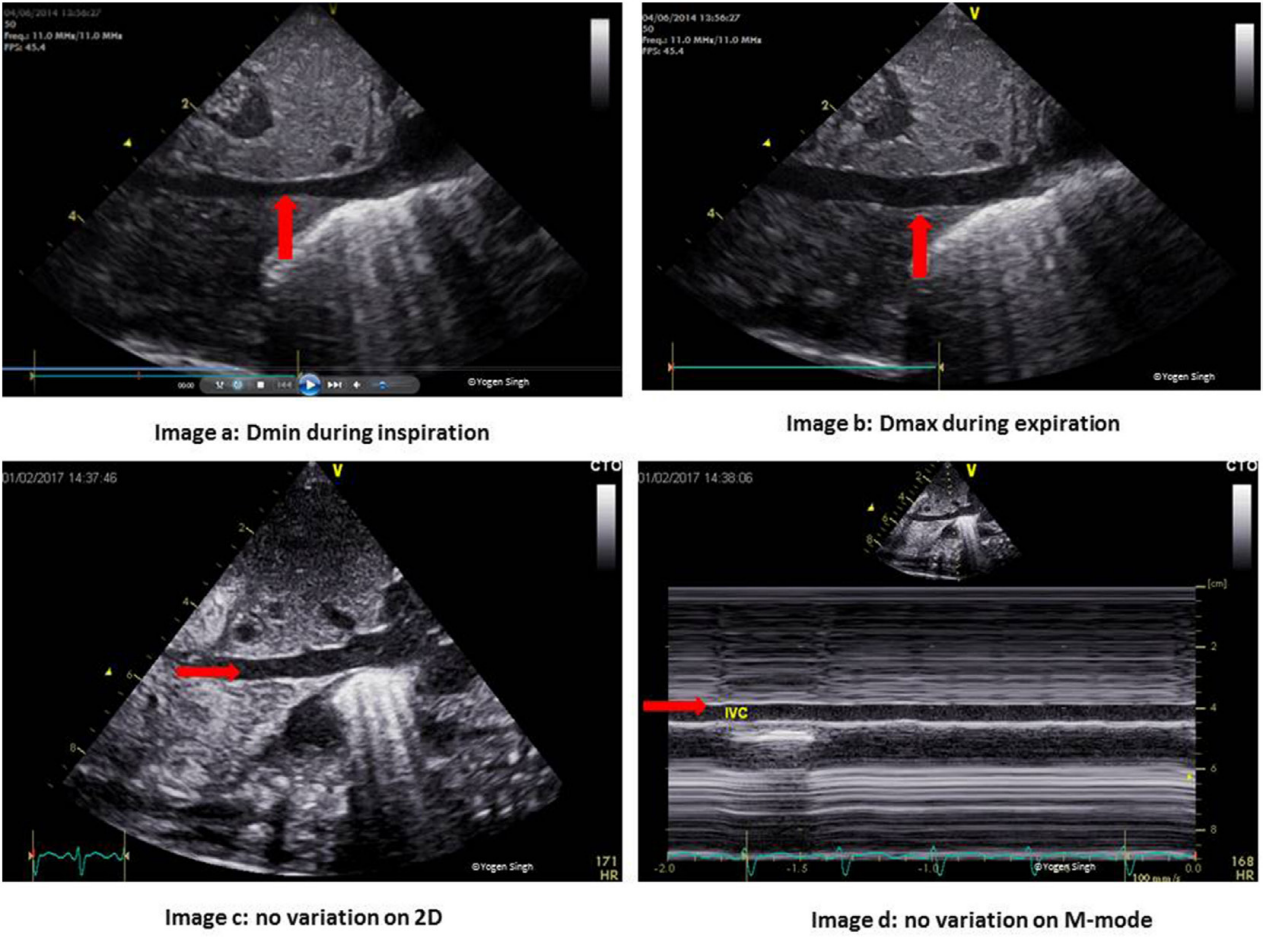
Inferior vena cava (IVC) changes during the respiratory and cardiac cycles. Images a,b show normal physiological change in the IVC diameter during inspiration and expiration. Images c,d show no variation during the respiratory cycle in the presence of volume loading of the heart.

Inferior vena cava distensibility index is calculated by measuring the Dmax and Dmin of IVC from the subcostal view (Figure 1), and IVCDI exceeding 18% has been reported to be predictive of fluid responsiveness in adults, and it is often extrapolated in children as well (32, 33):

$$\text{IVCDI}=\frac{\text{Dmax}-\text{Dmin}}{\text{Dmin}}.$$

However, one should be mindful that these methods are validated in self-breathing adults. They are difficult to assess in children on mechanical ventilation and their role in assessing fluid responsiveness in children needing mechanical ventilation remain uncertain. In author’s experience, these methods can help in guiding the fluid therapy in emergency situations, especially in the intensive care settings. Clinicians performing echocardiography should know their limitations and results should be interpreted in conjunction with other clinical parameters.

#### Assessment of Cardiac Function

The assessment of cardiac function and hemodynamic evaluation in sick children by performing physical examination is unreliable, even when it is performed by an experienced clinician (34). The studies have already established clear benefits of cardiac function on bedside echocardiography, and more importantly on the serial assessments longitudinally to review the clinical progress. The published evidence has demonstrated that targeted specific therapy was started earlier when the clinical decisions were made after acquiring additional physiological information by bedside point-of-care echocardiography, and this has been reported to change in the management in up to 37–60% cases (4, 11, 35).

A comprehensive review of the cardiac function is out of the scope of this article. We will review the common echocardiographic parameters used in assessing cardiac function in children, especially in the intensive care setting.

## Assessment of Left Ventricular Function

It has been suggested that echocardiographic analysis of LV function should be part of hemodynamic assessment in children with shock, and it should be used in conjunction with clinical parameters and physical examination (12). Ranjit et al. (10) have demonstrated a favorable outcome in >91% cases of shock when echocardiography was used for hemodynamic monitoring in the pediatric intensive care setting. Similarly, neonatologist-performed targeted echocardiography has been reported to improve the outcomes after patent ductus arteriosus (PDA) ligation by identifying the infants with LV dysfunction (post-PDA ligation syndrome), and a specific therapy was commenced earlier when bedside echocardiography was used in the clinical practice (4, 10, 36).

The qualitative assessment of LV contractility may be quickly performed by “eyeballing” by an experienced clinician from the apical four-chamber view, parasternal long axis view (PLAX), parasternal short axis view (PSAX), or subcostal view (8, 15). This information can, and probably should be, supplemented by the objective assessment of left ventricular function. It is important to use several windows to assess LV as no single view can provide a comprehensive picture of contractility.

The common echocardiographic parameters used for quantitative assessment of LV function in children are as follows: fraction shortening (FS), ejection fraction (EF), Doppler pattern of LV filling (E and A waves at mitral valve), and tissue Doppler imaging (TDI). Some of the newer techniques such as speckle tracking, strain rate, and 3-D imaging have been reported to be more accurate in assessing cardiac function. However, these parameters may need longer time and special equipment making them less useful for the clinical practice in the intensive care setting.

### EF and FS

**Figure 2:**
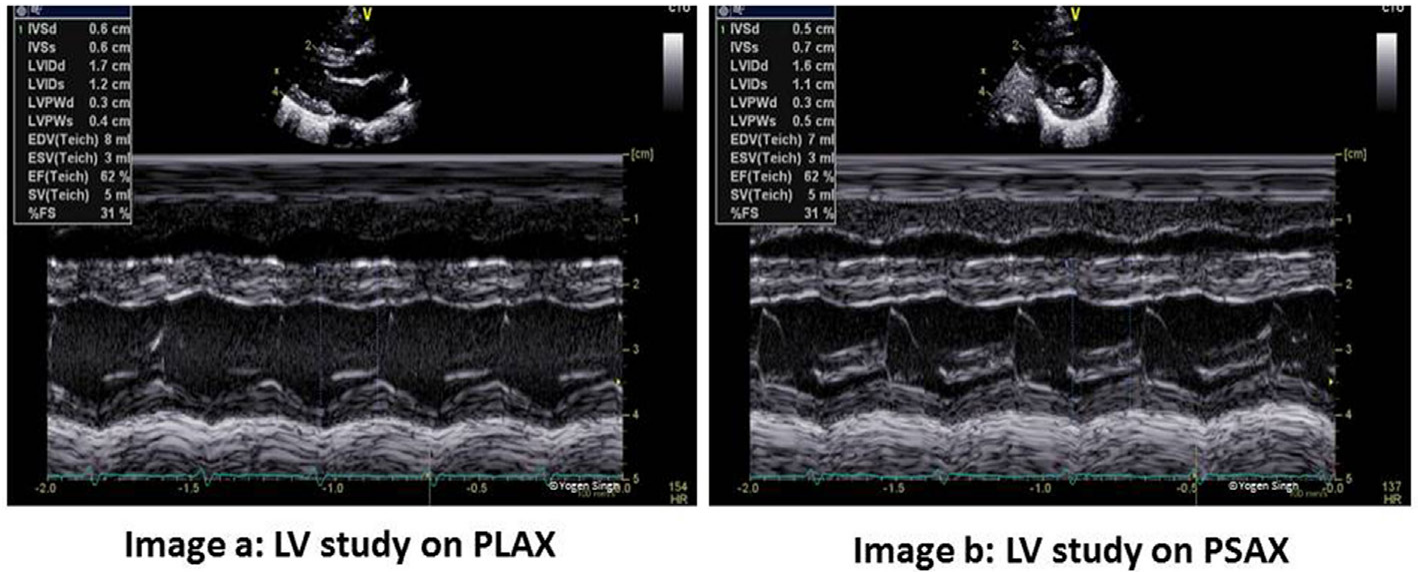
Left ventricle (LV) study in PLAX and PSAX views. FS and EF can be measured reliably on both views - image a shows FS measurement in PLAX and image b shows same measurement in PSAX view. However, EF probably best measured by Simpson’s method. PLAX, parasternal long axis view; PSAX, parasternal short axis view; LV, LV study; FS, fraction shortening; EF, ejection fraction.

Fraction shortening and EF can be calculated using M-mode in PLAX or PSAX views (Figure 2) or using Simpson’s biplane method. FS is calculated by measuring LV end-diastolic diameter (LVEDD) and LV end-systolic diameter (LVESD):

$$\text{Fraction shortening }(\text{FS})=\frac{\text{LVEDD}-\text{LVESD}}{\text{LVEDD}}\times 100\%.$$

Fraction shortening has been shown to correlate well with alterations in ventricular function related to preloading conditions following arterial switch operation (37).

**Figure 3:**
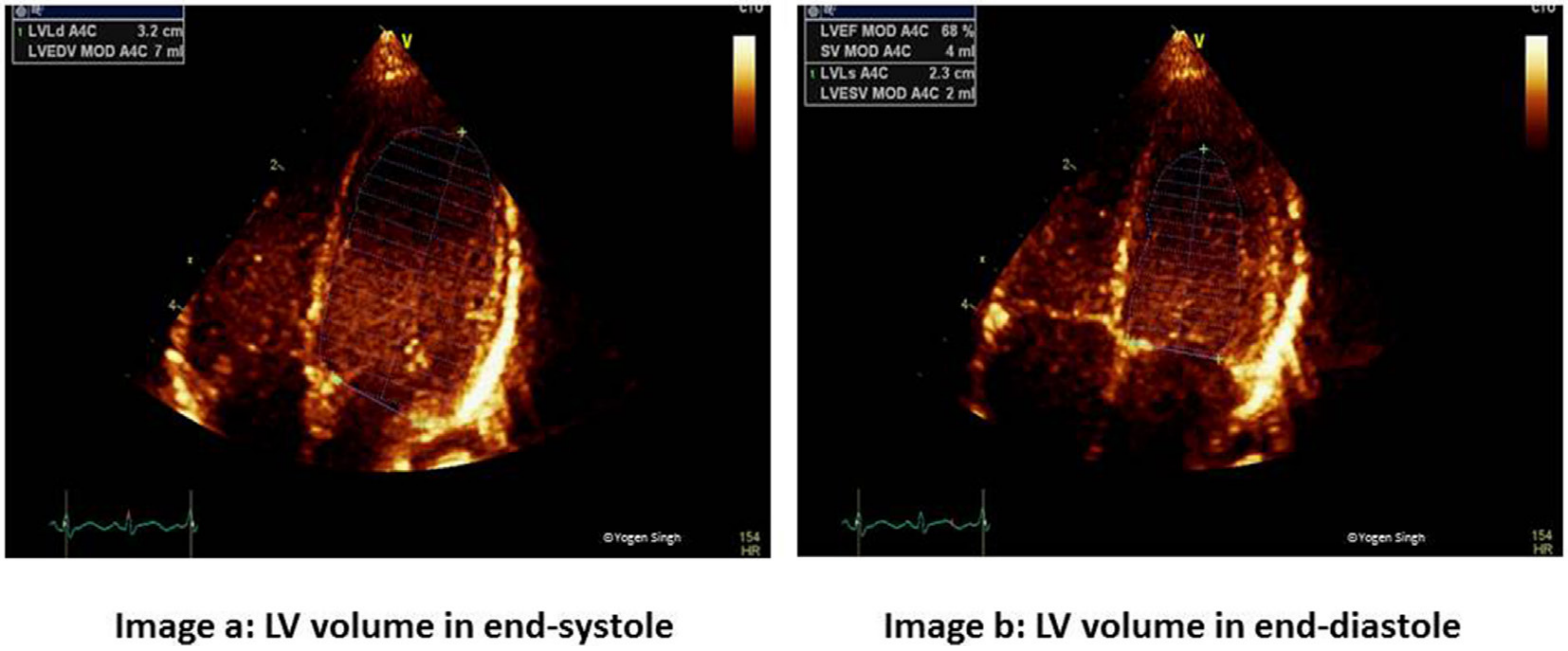
Simpson’s biplane method for measurement of ejection fraction (EF). Image a shows left ventricular end-diastolic volume (LVEDV) and image b shows left ventricular end-systolic volume (LVESV) measurements in apical four-chamber view to calculate EF. Similar measurements are done in apical two-chamber view.

Ejection fraction reflects stroke volume assessed on echocardiography. It can be calculated by using any echo mode, such as 2D, M-mode, and 3-D mode, which can delineate LV endomyocardial lining at end-systole and end-diastole. The recommended method for EF calculation is the Simpson’s or modified Simpson’s method (38) using apical four-chamber and apical two-chamber views (Figure 3). The LV cavity is divided into cylinders or disks, and uses the radius and length of the multiple disks measured to calculate a LV end-diastolic volume and LV end-systolic volume. This may be challenging in sick neonates and young children needing mechanical ventilation where windows can be poor and delineation of endocardium may be sub-optimal. Relative tachycardia and non-elliptical LV shape in ICU setting may give inaccurate measurements. Despite these limitations, EF is preferred over FS as it accounts images for regional wall motion abnormalities, and in most children, images are easy to acquire (38).

Ejection fraction has been studied to assess LV function immediately following cardiopulmonary bypass, and it has correlated well with invasive measures of LV function longitudinally. The clinical utility of EF in pediatrics has been established in multiple studies (39, 40). The normal values for EF and FS are well established in neonates and children. Normal FS is between 26 and 46%, while EF is classified as normal when EF is >55%, slightly reduced (EF 41–55%), moderately reduced (EF 31–40%), and markedly reduced (EF 30%) (10, 41).

The image acquisition for assessment of FS and EF is rapid and easy, and the calculations are simple and easily reproducible making them good bedside echocardiographic parameters for use in emergency situations and in the routine clinical practice (9). The clinical usefulness of quantitative EF and FS measurements in the management of critically ill patients is broadly accepted in pediatric patients (9, 10, 41, 42). However, in addition to the challenges of image acquisition, they are dependent on preload and afterload, and also influenced by the ventricular geometry and interventricular septal deviation. This should be taken into account while making clinical decisions based upon EF and FS measurements, and it has been recommended to use them in conjunction with clinical parameters and other echocardiographic parameters (43). These are excellent tools to monitor the clinical progress by serial assessments longitudinally over time.

### Tissue Doppler Imaging

Tissue Doppler imaging allows quantitative assessment of regional and global myocardial function by detecting changes in myocardial deformation based on the tissue velocities (44, 45). During the cardiac cycle, atrioventricular valve moves toward apex in systole (myocardial contraction) and away from the apex during diastole (myocardial relaxation). This myocardial movement (longitudinal deformation) can be best assessed by interrogating longitudinal annular velocities of the atrioventricular valve and interventricular septum (IVS) using pulse-wave (PW) Doppler in apical four-chamber view. This allows examining any particular area of interest and hence assessing regional myocardial function. Myocardial deformation has been shown to correlate well with global and regional ventricular contractility (45–47).

**Figure 4:**
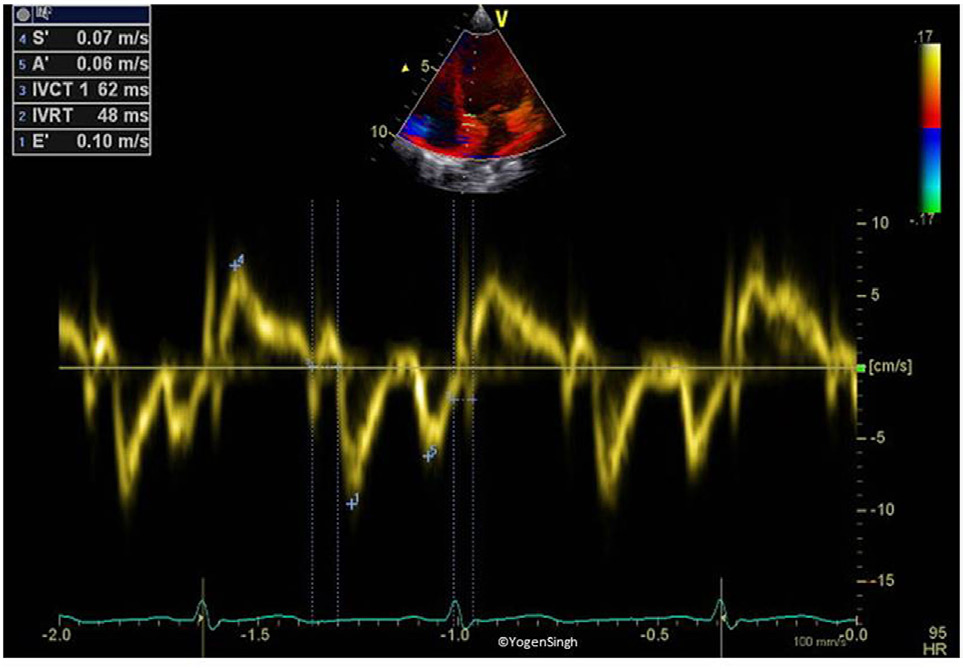
Tissue Doppler imaging on pulse-wave Doppler of interventricular septum (myocardium). Spectral above the baseline (s′) reflects the movement of myocardium toward apex in systole while below the baseline (e′ and a′) reflects the movement of myocardium away from apex in diastole.

Pulse-wave Doppler interrogation of the myocardium, rather than on blood flow, is performed, and hence, this Doppler study reflects low velocity signals on TDI. A Doppler trace of a small segment of myocardium or atrioventricular valve annulus is obtained. Using PW Doppler, a velocity waveform is generated—spectral above the baseline (s′) shows movement of myocardium during systole while e′ and a′ waves (below the baseline) show diastolic movement of myocardium (Figure 4).

Assessing myocardial function using TDI has several benefits in children. This is relatively independent of ventricular geometry and loading conditions. The image acquisition is easy and fast. The results are easily obtained on bedside without using any special software, which makes it an attractive echocardiographic parameter in the ICU setting. However, like all other Doppler techniques, TDI is highly dependent on the angle of insonation and emphasis should be on keeping it minimal by aligning ultrasound beam parallel to the myocardium while imaging acquisition.

## Assessment of RV Function and Pulmonary Hypertension

**Figure 5:**
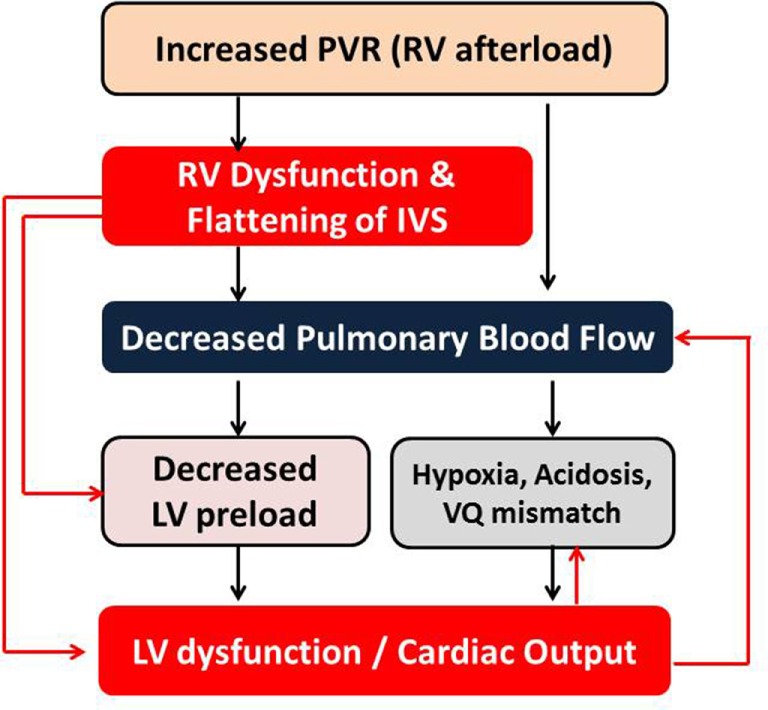
Interdependency of right and left ventricular functions in the setting of pulmonary hypertension with increased PVR. LV, left ventricle; RV, right ventricle; PVR, pulmonary vascular resistance; IVS, interventricular septum; VQ, ventilation–perfusion.

The assessment of right ventricular function is of particular interest to the intensivists because common interventions performed in the ICUs, such as mechanical ventilation and fluid therapy, often affect pulmonary vascular resistance and RV function. The persistent pulmonary hypertension of newborn (PPHN) is a common condition affecting infants in the neonatal ICU. The echocardiographic assessment of pulmonary hypertension is one of the most immediate uses of bedside functional echocardiography in the intensive care setting, and it helps in providing targeted specific therapy and monitoring response to such intervention. Due to the ventricular interdependence, impaired RV function may lead to LV dysfunction (Figure 5) and *vice versa* (48, 49). The published studies have demonstrated that right ventricular dysfunction is associated with poor outcome in infants with PPHN and congenital diaphragmatic hernia (50, 51). The right ventricular failure is independently associated with increased mortality in critically ill patients (52).

The shape of the RV makes RV function assessment more challenging, even in the expert hands. Recently, there has been a great interest in assessing RV function in sick children. Similar to LV function assessment, RV function can be quickly assessed from its size, wall thickness, and contractility by “eyeballing” (qualitative assessment). Apical four-chamber view provides good overview of RV contractility, assessment of RV and RA size, and presence of tricuspid regurgitation (TR). However, it should be examined in multiple views PLAX, PSAX, and sub-costal views to further assess interventricular septal movements, RV wall thickness, and RV dimensions. Direct measurement of RV size is not recommended due to its complex geometry and the presence of trabeculations within the RV cavity. Traditionally RV systolic function was assessed qualitatively only. However, newer techniques such as TDI and tricuspid annular plane systolic excursion (TAPSE) can be used to assess RV function reliably in children, even in the intensive care setting (41, 42, 53).

Comprehensive guidelines for the echocardiographic assessment of the right heart are given in a recent report of the American Society of Echocardiography (54). Similar guidance has been published for the assessment of RV function in neonates (42). A full comprehensive assessment of RV function is out of the scope of this review article, and hereafter, we will focus on the echocardiographic assessment of pulmonary hypertension and RV function assessment in children, especially on the echo parameters particularly useful in the intensive care setting. The most useful echocardiographic parameters to assess the RV function and pulmonary hypertension quantitatively in children are as follows: estimation of PAP, assessment of ductal or atrial shunt, assessment of IVS and LV shape, TAPSE, and pulmonary artery acceleration time (PAAT).

### Estimation of PAP

**Figure 6:**
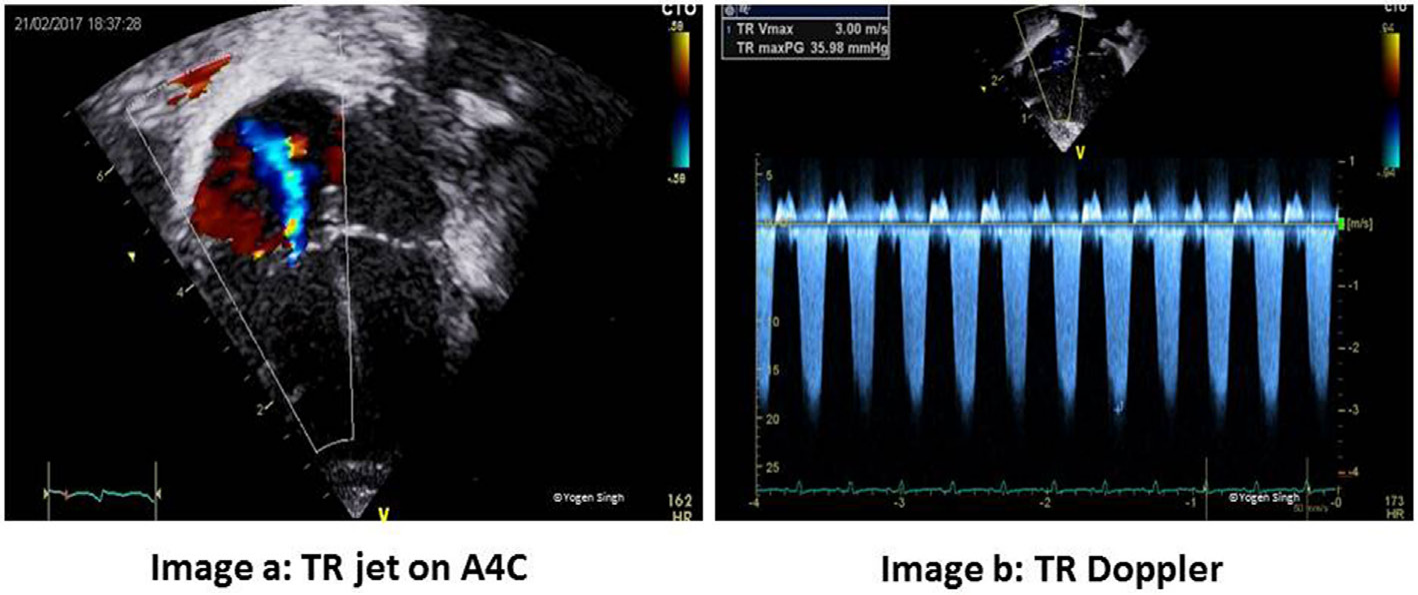
Estimation of pulmonary artery systolic pressure (PASP). Image a shows tricuspid regurgitation (TR) jet in apical four-chamber view (A4C) and image b with Doppler assessment of TR—in this case a gradient of around 36 mmHg between right ventricle and right atrium (RA) suggests PASP between 40 and 45 mmHg (36 + RA pressure of 5–10 mmHg).

The pulmonary artery systolic pressure (PASP) can be reliably estimated using echocardiography in the presence of TR. Using the continuous-wave (CW) Doppler, the pressure gradient between RV and RA is calculated by using modified Bernoulli’s principle (41, 42, 51, 55–58). This is best measured in the apical four-chamber view or PLAX sweep view to obtain good trace of TR while keeping the angle of insonation minimal. RA pressure, which is usually between 5 and 10 mmHg, is added to this peak pressure gradient obtained on CW Doppler to obtain PASP, when there is no RV outflow tract obstruction (Figure 6):

$$\begin{array}{l} \text{Pulmonary artery systolic pressure}\\ \;=4\times {\left(\text{TR velocity}\right)}^{2}+\text{RA pressure }(5-10\text{ mmHg}). \end{array}$$

The mean PAP can be estimated by measuring the pulmonary regurgitant velocity at the start of pulmonary regurgitation when present.

### Assessment of Ductal and Atrial Shunt

The presence of ductal and/or atrial shunt can provide a quick guide whether PA pressure is higher than systemic pressure, equal, or lower. The visual inspection on color flow Doppler or PW/CW Doppler can help in assessing the direction of blood flow. The right to left ductal shunt suggests a supra-systemic PAP, while bi-directional shunt suggests that PAP is equal to systemic artery pressure. A left to right shunt is reflective of PAP lower than systemic pressure. In PPHN, atrial shunt [across persistent foramen ovale (PFO)/atrial septal defect (ASD)] is often bi-directional. In presence of a pure right to left shunt, total anomalous pulmonary venous drainage should be excluded until proven otherwise.

### Assessment of IVS and LV Contour

**Figure 7:**
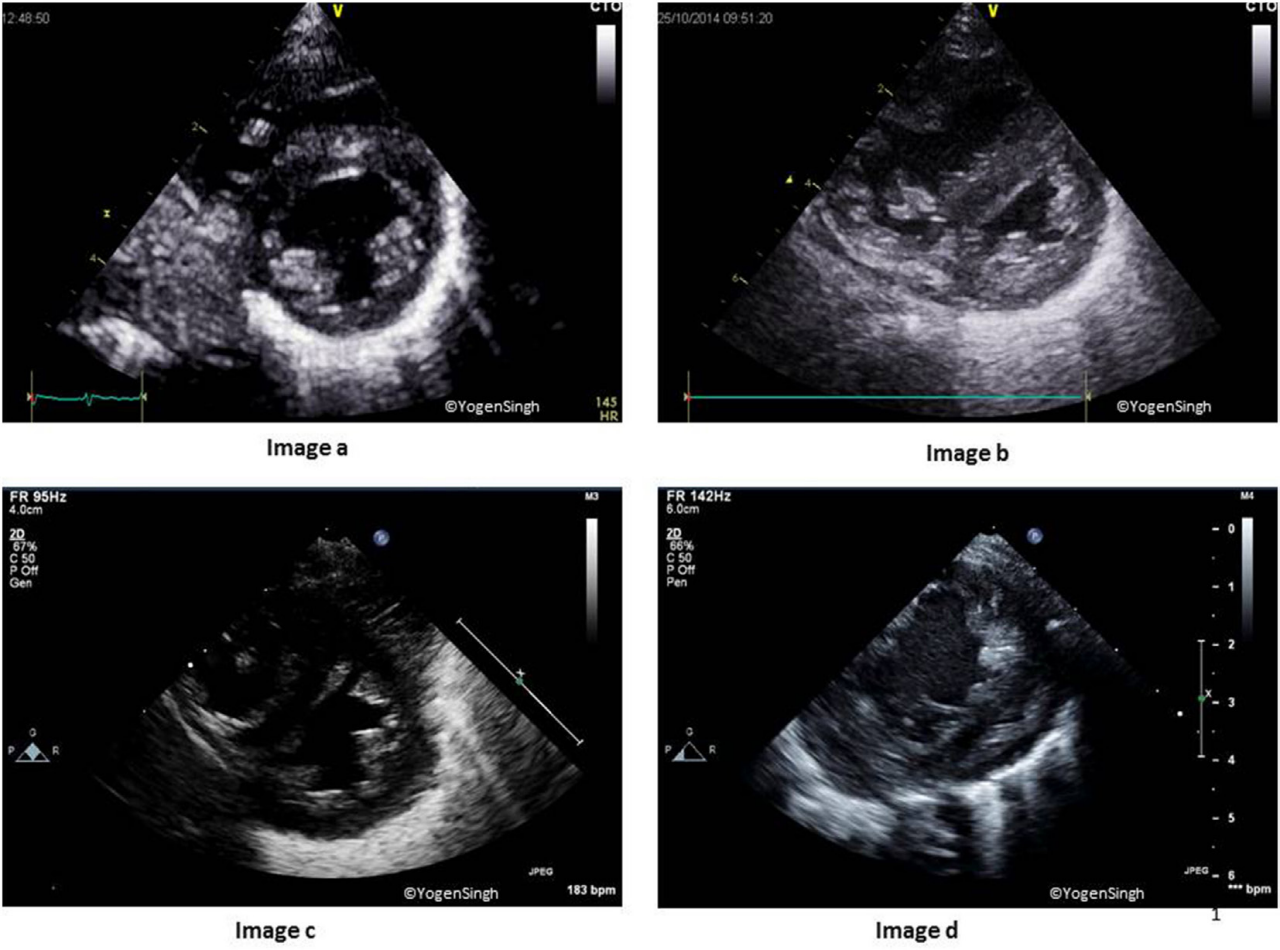
Interventricular septum (IVS) and left ventricle (LV) shapes in pulmonary hypertension. Image a shows normal IVS and LV shape with LV being a circular-shaped structure on sweep PSAX view. Images b–d show change in shapes with increasing flattening of IVS in presence of mild, moderate, and severe pulmonary hypertension, respectively.

This is best assessed by using PSAX sweep views. Normally, LV is seen as a circular structure in PSAX view because LV pressure is much higher than the RV pressure. In presence of pulmonary hypertension, there may be flattening of IVS, making LV a D-shaped structure. The severity of flattening or reversal of IVS reflects severity of pulmonary hypertension as shown in Figure 7 (38, 42).

### RV to LV Ratio

Normally, LV size is bigger than RV in children and adults. RV/LV ratio of more than 1 suggests pulmonary hypertension, and it is best calculated in the PSAX view on 2D mode (38, 42).

### Tricuspid Annular Plane Systolic Excursion

**Figure 8:**
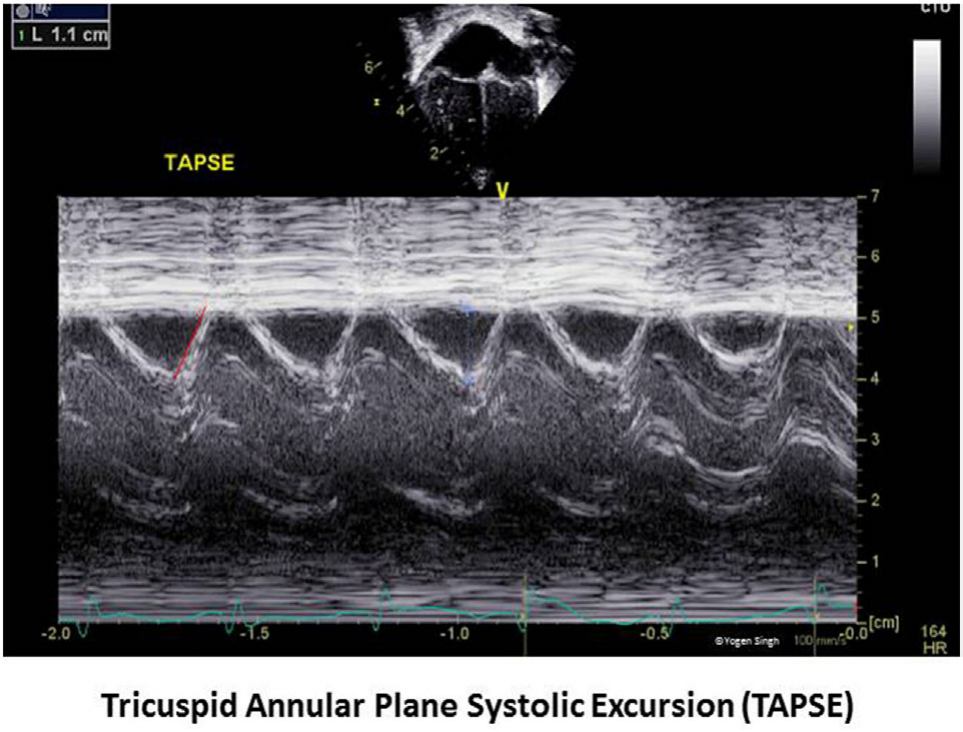
Tricuspid annular plane systolic excursion (TAPSE) measurement on echocardiography. TAPSE reflects the longitudinal movement of the tricuspid annulus toward apex in systole measured on M-mode in apical four-chamber view.

Tricuspid annular plane systolic excursion reflects systolic displacement of the tricuspid annulus toward the RV apex along the longitudinal axis, and it closely correlates with RV EF. TAPSE is usually acquired by placing the M-mode cursor through the lateral tricuspid annulus and measuring the amount of longitudinal motion of the annulus in peak systole (Figure 8). There is paucity of published evidence in children. Lammers et al. reported that TAPSE and PAAT are good predictors of RV systolic function (59). The assessment of TAPSE has several benefits—it can be easily measured on echocardiography in real time without need of any offline assessment or special software. Even more importantly, TAPSE is not dependent on RV geometry and is less influenced by imaging artifacts, which makes it a very useful maker in clinical practice in children (60). However, care should be taken to align the cursor line with the myocardial movement as Doppler assessment is angle dependent.

### PAAT and PAAT/Right Ventricular Ejection Time (RVET) Ratio

**Figure 9:**
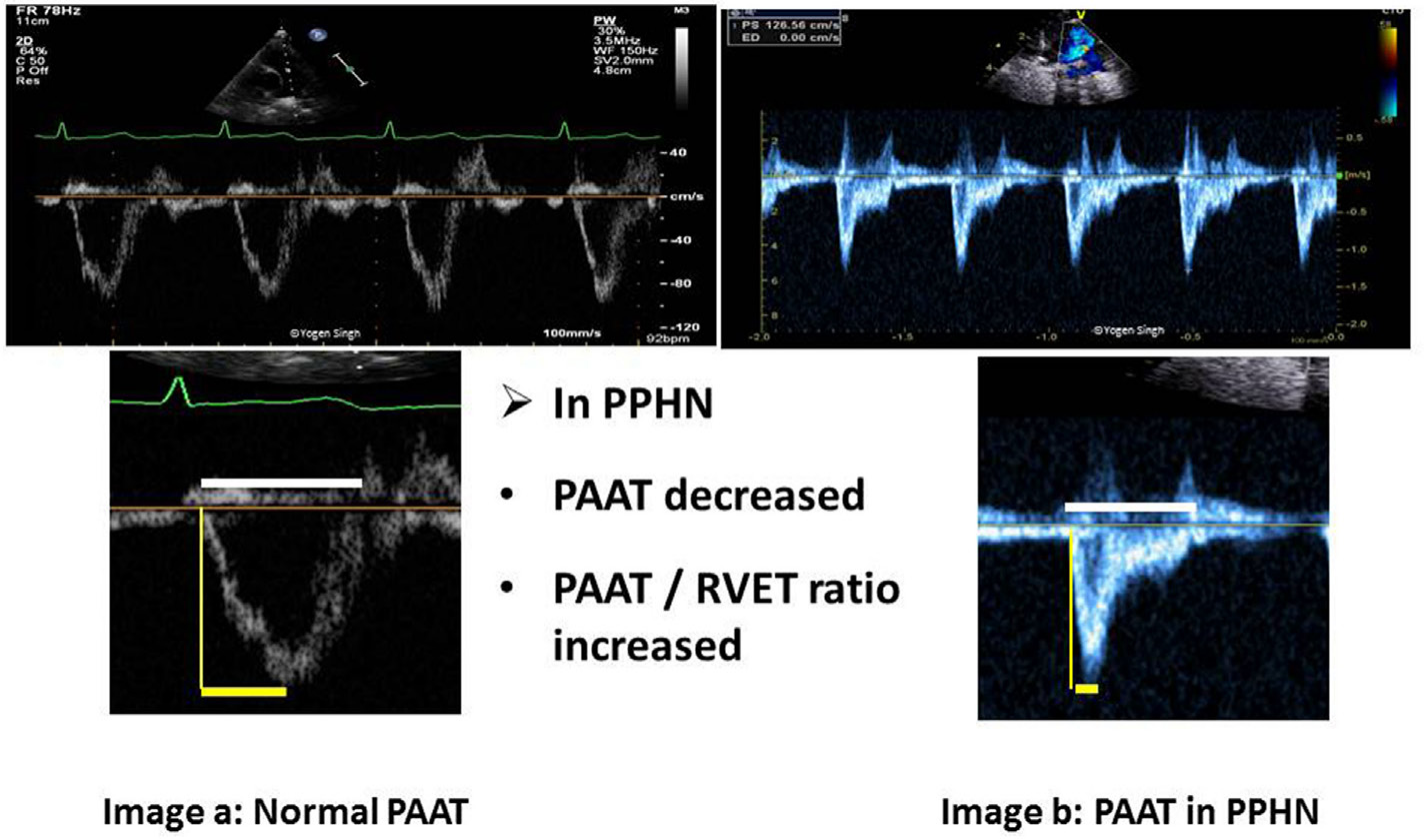
Pulmonary artery acceleration time (PAAT) measurement. Image a shows normal ratio of PAAT and right ventricular ejection time (RVET) in a normal child. Image b shows significantly decreased PAAT and increased PAAT/RVET ratio suggestive of pulmonary hypertension. “Dicrotic notch” may be seen on pulse-wave Doppler spectral in the pulmonary artery.

A quick visual inspection of pulmonary artery Doppler spectral showing “dicrotic notch” may suggest presence of pulmonary hypertension. However, this is a qualitative test with low sensitivity and specificity, and it may not be seen even in severe pulmonary hypertension. The PAAT can be easily calculated from PW spectral of pulmonary artery (Figure 9) and PAAT <90 ms in neonates is highly predictive of pulmonary hypertension. PAAT value varies with age of the child and HR. In older children, PAAT of <110 ms reflects pulmonary hypertension. A ratio of PAAT/RVET can be easily calculated, and a value of <0.31 suggests pulmonary hypertension with a sensitivity and specificity above 90% (61). Recently, pediatric reference values for PAAT were published (62).

## Assessment of Cardiac Output and Blood Flow on Echocardiography

The echocardiographic assessment of blood flow across any “vessel or outflow tract” can be performed by multiplying the cross-sectional area (CSA) of the vessel by the VTI of blood flow across specific point where CSA and HR are calculated, and applying these values in the equation below (63, 64):

$$\text{Blood flow }\left(\text{ml}/\text{kg}/\text{min}\right)=\frac{\text{CSA}\times \text{VTI }\left(\text{in cm}\right)\times \text{HR}}{\text{Body weight }\left(\text{in kg}\right)}.$$

This principle can be applied to calculate the blood flow across any blood vessel or outflow tract. It is being applied in the clinical practice to estimate left ventricular output (LVO), right ventricular output (RVO), and superior vena flow in children. Blood flow and cardiac output are not interchangeable.

### Estimation of Left Ventricular Output

**Figure 10:**
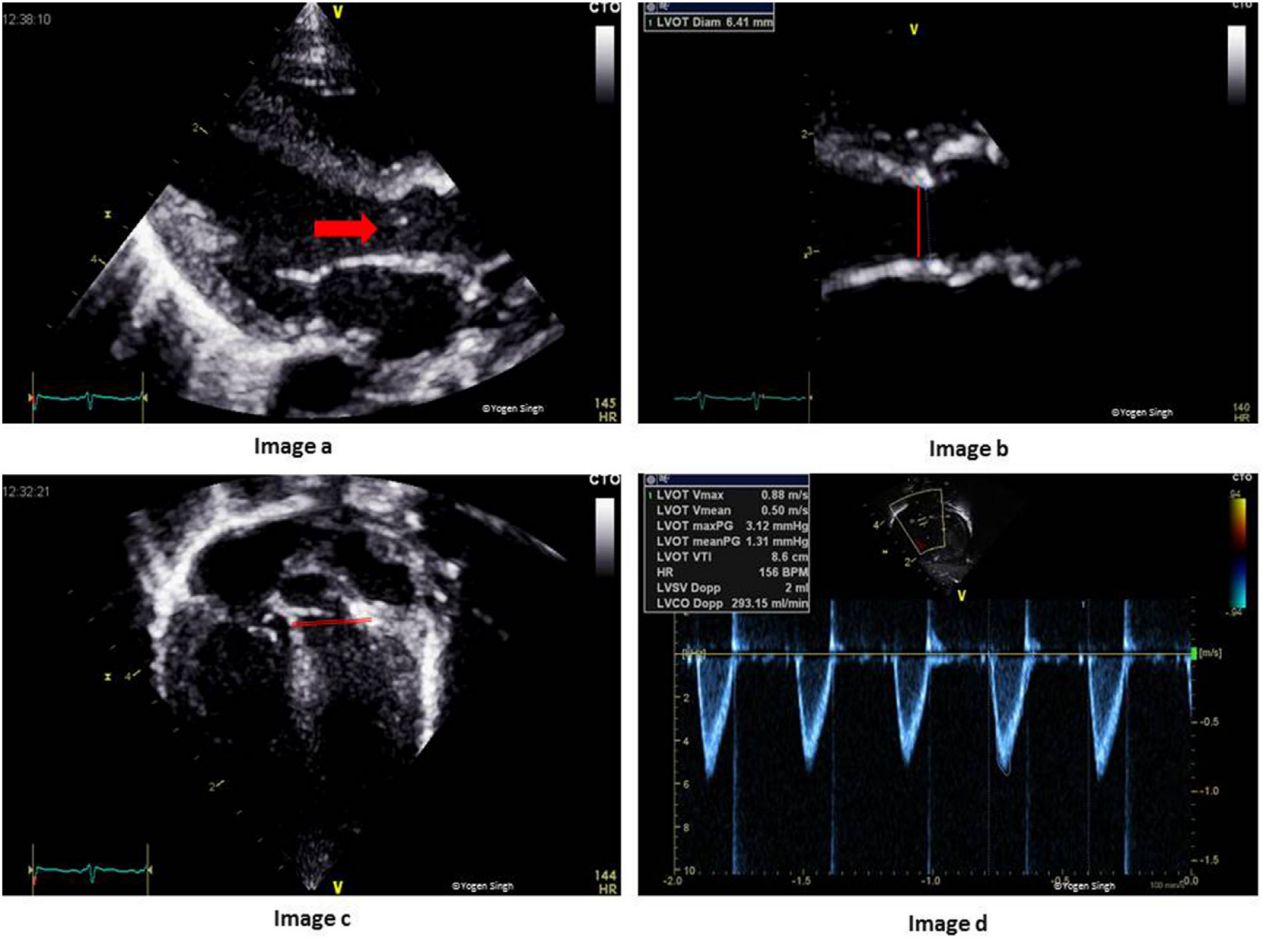
Assessment of left ventricular output (LVO) on echocardiography. Image a shows LV outflow tract (red arrow) and AV annulus (red line), which has been zoomed in image b to measure diameter at the hinge point of AV valve. Image c shows LV outflow tract and the site for PW Doppler to measure VTI (red line showing PW sample gate). Image d shows LVO in milliliters per minute. AV, aortic valve; PW, pulse wave; LV, left ventricle; VTI, velocity time integral.

The systemic blood flow equals LVO in the absence of cardiac shunts. The CSA is calculated by measuring the diameter at the hinge point of AV annulus at end-systole in the PLAX, and VTI is measured just proximal to the AV valve by using PW Doppler in the apical five-chamber view (Figure 10). HR is calculated automatically by the ultrasound machine from the ECG recording:

$$\begin{array}{l} \text{Left ventricular output }(\text{ml}/\text{kg}/\text{min})\\ \;=\frac{\text{CSA }(\text{at AV annulus})\times \text{VTI}-\text{LVOT }(\text{in cm})\times \text{HR}}{\text{Body weight }(\text{in kg})}. \end{array}$$

Despite various assumptions and limitations (discussed below), the assessment of LVO on echocardiography correlate strongly with the measurements acquired by other well-established techniques such as pressure measurement by cardiac catheter and Fick’s dye dilution method. The published studies showed a bias under 10%. In a recently published study, left ventricular cardiac output estimate on echocardiography correlated strongly with the results from phase contrast MRI, which is very reassuring (27, 65).

### Estimation of RVO

**Figure 11:**
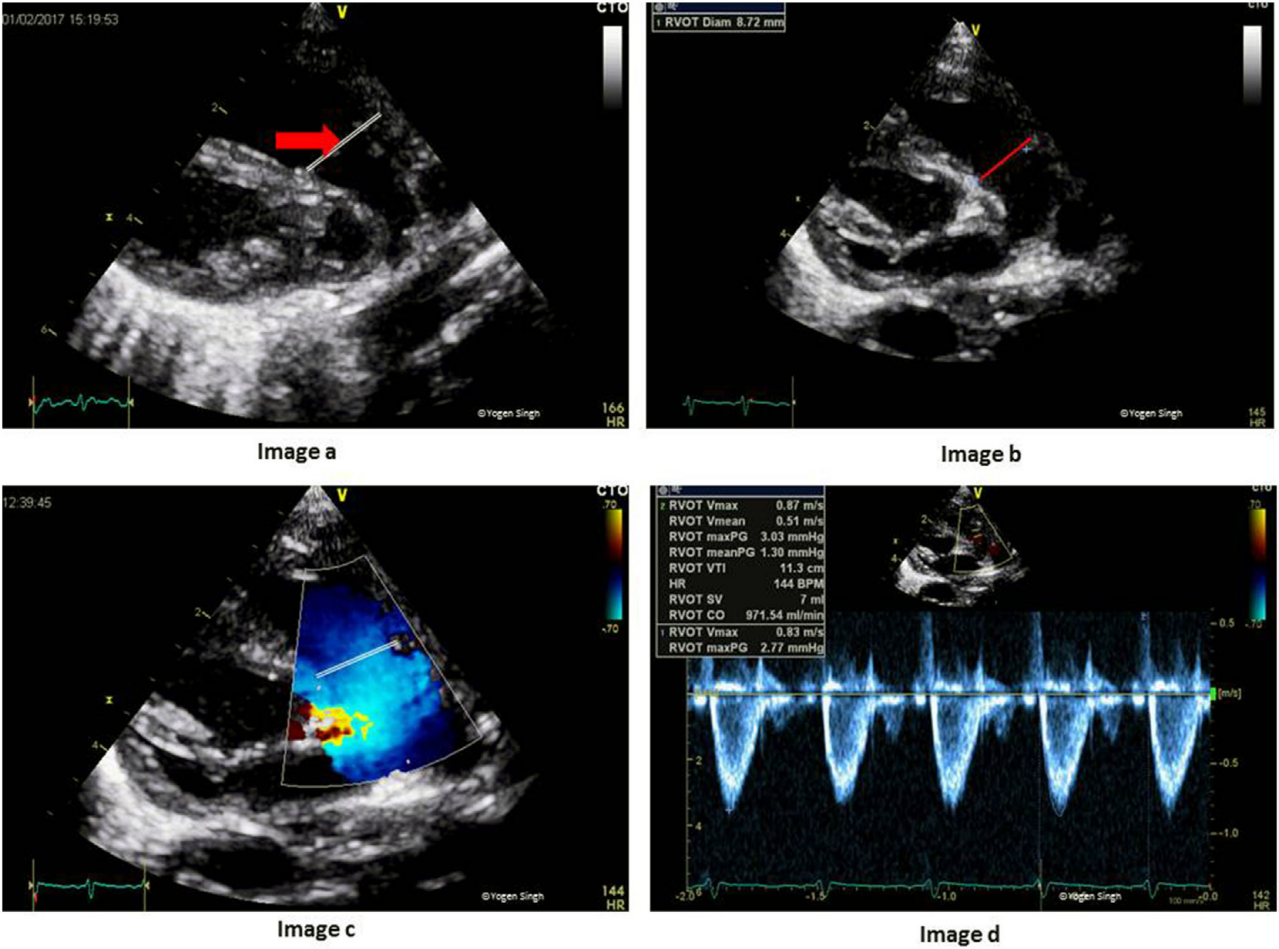
Assessment of right ventricular output (RVO) on echocardiography. Image a shows RV outflow tract (red arrow) and image b with measurement of PV annulus (red line) at the hinge point of PV valve. Image c shows RV outflow tract and the site for PW Doppler to measure VTI (red line showing PW sample gate). Image d shows RVO in milliliters per minute. PV, pulmonary valve; PW, pulse wave; RV, right ventricle; VTI, velocity time integral.

The RV output equals to systemic venous return in the absence of cardiac shunts. RV output can be easily assessed on echocardiography. The CSA is calculated by measuring diameter at the hinge point of pulmonary valve (PV) annulus at end-systole in the parasternal long axis sweep view (PLAX) or PSAX, and VTI is measured just proximal to the PV by using PW Doppler in the same views (Figure 11). HR is calculated automatically by the ultrasound machine from the ECG recording:

$$\text{Right ventricular output }(\text{ml}/\text{kg}/\text{min})=\frac{\text{CSA }(\text{at PV annulus})\times \text{VTI}-\text{RVOT }(\text{in cm})\times \text{HR}}{\text{Body weight }(\text{in kg})}.$$

#### Limitations of LVO and RVO Measurement Using Echocardiography

The measurement of LVO and RVO using echocardiography has the following limitations:

(1) LVO and RVO assessment is contaminated by presence of shunts (shunt across ductus arteriosus or atrial shunt), which are quite common in neonates and in children with congenital heart defects. In the presence of patent ductus arteriosus (PDA), left ventricular output reflects systemic blood flow plus the amount of ductal shunt. Similarly, in the presence of PFO or ASD, RVO reflects the systemic blood flow plus atrial shunt volume. The presence of other septal defects such as ventricular septal defect or ASD will result in further over-estimation of LVO or RVO;
(2) Measurement of CSA is prone to mistakes, especially in neonates and young children. As CSA is calculated by squaring the diameter, any error in measurement is multiplied;
(3) Measurement of VTI/stroke distance is also prone to errors. The angle of insonation, angle between the Doppler cursor and the blood flow, should be minimal. If the angle of insonation is >5–10%, it would under-estimate the cardiac output. Moreover, calculation of VTI needs manual tracing of PW Doppler spectral which also carries the risk of error in measurement; and
(4) Intra-observer variability on the echocardiographic assessment of blood flow is quite high. The published studies have reported an intra-observer variability of 12 and 22% for RVO and LVO assessments, respectively (66, 67). The inter-observer variability is even higher.

The echocardiographer should be aware of these limitations and precautions should be taken to minimize such errors.

#### Application of LVO and RVO Measurements in the Clinical Practice

Despite the limitations discussed above, assessment of left and RVO is reliable in children without shunts, and even in neonates, it is fairly reliable in expert hands. The authors find the trend on serial echocardiography more useful than the absolute value in clinical practice—CSA for any patient does not change and HR can be calculated precisely at any time. Hence, serial assessment of VTI may help in studying the impact of any intervention on cardiac output in real time.

### Superior Vena Cava (SVC) Blood Flow

Superior vena cava flow can be assessed on echocardiography using an approach similar to RVO and LVO measurements. SVC flow has been proposed as a surrogate measure of cerebral blood flow, and it has been associated with short-term and long-term outcomes in neonates (68, 69). No doubt, this generated a lot of interest in studying SVC flow and its hemodynamic impact in the neonatal period. Although it has been proposed as a surrogate marker of cerebral blood flow, it also reflects venous return from upper part of the body. Several studies have reported an association between low SVC flow in the first 24 h and intraventricular hemorrhage and/or neonatal death in preterm infants (68, 69). However, other studies could not demonstrate such association (23).

**Figure 12:**
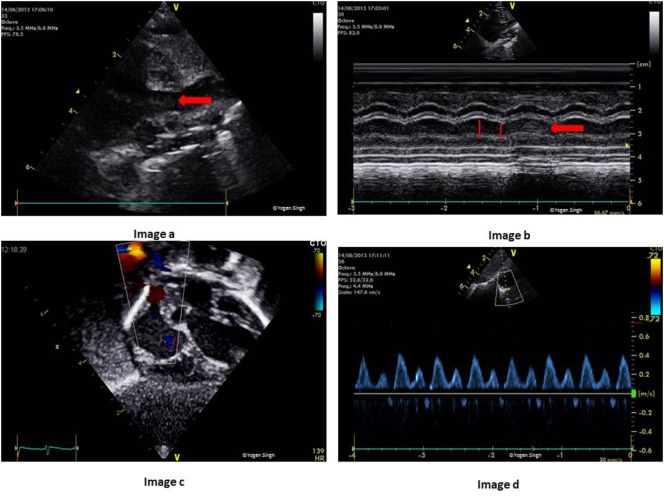
Measurement of superior vena cava (SVC) flow on echocardiography. Image a shows SVC in modified parasternal short axis view on 2D and image b with measurement of SVC diameter in M-mode. Image c shows acquisition of pulse-wave Doppler in the sub-costal view and SVC flow in milliliters per minute has been calculated in image d after measuring velocity time integral.

For the measurement of SVC flow, CSA is calculated by measuring SVC diameter in modified PSAX view at the level of right pulmonary artery, and VTI is measured just proximal to its connection to RA by using PW Doppler in the sub-costal or right suprasternal view (Figure 12). The SVC is a venous structure which is D-shaped and collapsible. Hence, measuring CSA of SVC is prone to increased error as compared to a relatively non-collapsible AV or PV annulus. It is recommended that SVC diameter should be averaged over 5–10 heart cycles and best measured in M-mode. Similarly, VTI is also averaged over 5–10 cardiac cycles. HR is calculated automatically by the ultrasound machine from the ECG recording:

$$\text{SVC blood flow }(\text{ml}/\text{kg}/\text{min})=\frac{\text{CSA }(\text{SVC in M-mode})\times \text{VTI }(\text{in cm})\times \text{HR}}{\text{Body weight }(\text{in kg})}.$$

The reference values for SVC flow in term and preterm infants have been published with considerable variations (24–26). Given the risk of errors in measuring SVC diameter accurately, there is no surprise that the validation studies on estimating SVC blood flow by using echocardiography in neonates showed poor correlation when compared to MRI (27). The studies have reported high intra- and inter-observer variability, a significant bias and an error percentage of up to 55% (24–27). In a recently published study using a modified approach to measure SVC flow, VTI measured from suprasternal view and CSA calculated by tracing SVC area in PSAX view at the level of right pulmonary artery, the authors compared SVC flow on echocardiography with the results from the phase contrast MRI. They reported an improvement in the accuracy and repeatability of SVC flow measurement using this modified technique; however, there remained a bias of 17.7% and an error percentage of 36.9 (28). With the current published evidence, it is hard to recommend using SVC flow in clinical decision making. However, like RVO and LVO, a trend in SVC flow can be useful in clinical practice to assess the impact of any intervention.

## Conclusion

Echocardiography remains the investigation of choice for diagnosing congenital heart effects in children. Traditionally used clinical parameters (HR, BP, respiration rate, oxygen saturation, urine output, and serum lactate) are surrogate markers of cardiovascular well-being. Functional echocardiography has the ability to provide direct evaluation of hemodynamic status in children. Bedside echocardiography is easily available. It is safe and non-invasive, which makes it an attractive tool for hemodynamic assessment in the intensive care setting.

The point-of-care echocardiography is now increasingly being used to assess cardiac function, fluid responsiveness, estimate PAP in pulmonary hypertension, provide specific intervention and monitor response to treatment, and choose inotropes or vasopressors based upon echocardiographic findings. Bedside echocardiography should be considered as an extension of physical examination, and it should be used in conjunction with the existing clinical parameters in making clinical decisions. The physiological information acquired by echocardiography may help the intensivists in making a timely, accurate, and appropriate diagnosis. However, clinicians should know the limitations of these parameters needing high-quality images for accurate measurement, which can be a challenge in the intensive care setting even in the expert hands. There is an urgent need of a structured training program for the intensivists to learn echocardiography skills, which can be used safely and effectively in the intensive care setting.

## Author Contributions

The author confirms being the sole contributor of this work and approved it for publication.

## Conflict of Interest Statement

The author declares that the research was conducted in the absence of any commercial or financial relationships that could be construed as a potential conflict of interest.
